# A study on EEG feature extraction and classification in autistic children based on singular spectrum analysis method

**DOI:** 10.1002/brb3.1721

**Published:** 2020-10-30

**Authors:** Jie Zhao, Jiajia Song, Xiaoli Li, Jiannan Kang

**Affiliations:** ^1^ Institute of Electronic Information Engineering Hebei University Baoding China; ^2^ Machine Vision Technology Creation Center of Hebei Province Baoding China; ^3^ State Key Laboratory of Cognitive Neuroscience and Learning Beijing Normal University Beijing China

**Keywords:** alpha peak frequency (APF), autism, classification, electroencephalography, singular spectrum analysis (SSA)

## Abstract

**Introduction:**

The clinical diagnosis of Autism spectrum disorder (ASD) depends on rating scale evaluation, which introduces subjectivity. Thus, objective indicators of ASD are of great interest to clinicians. In this study, we sought biomarkers from resting‐state electroencephalography (EEG) data that could be used to accurately distinguish children with ASD and typically developing (TD) children.

**Methods:**

We recorded resting‐state EEG from 46 children with ASD and 63 age‐matched TD children aged 3 to 5 years. We applied singular spectrum analysis (SSA) to the EEG sequences to eliminate noise components and accurately extract the alpha rhythm.

**Results:**

When we used individualized alpha peak frequency (iAPF) and individualized alpha absolute power (iABP) as features for a linear support vector machine, ASD versus TD classification accuracy was 92.7%.

**Conclusion:**

This study suggested that our methods have potential to assist in clinical diagnosis.

## Graphical abstract

We sought biomarkers from resting‐state electroencephalography (EEG) data that could be used to accurately distinguish ASD children and typically developmental (TD) children. When we used individualized alpha peak frequency (iAPF) and individualized alpha absolute power (iABP) as features for a linear support vector machine, ASD versus TD classification accuracy was 92.7%, suggesting that our methods have potential to assist in clinical diagnosis.

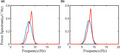

## 1 INTRODUCTION

Autism spectrum disorder (ASD) is a serious neurodevelopmental disorder with core symptoms which include communication and social disorders and stereotyped behavior (Chez et al., 2006). The current incidence of ASD is rising rapidly around the world. The cause of ASD is presently unclear, and its diagnosis is mainly based on rating scales and behavioral observation, which are subjective. Therefore, it is worthwhile to find objective indicators for accurate assessment.

Electroencephalography (EEG) is a powerful tool for analyzing and evaluating brain development due to its millisecond time resolution (Ingber & Nunez, 2011). The frequency domain of resting‐state EEG recordings is generally divided into four bands: delta band, theta band, alpha band, and beta band (Akin, 2002). Alpha band oscillation is one of the most significant dynamic features of the brain in resting and awake states (Fan, 2018; Klimesch, Sauseng, & Hanslmayr, 2007). Most studies define alpha band as a fixed narrowband (commonly 8–12 Hz) (Haegens, Cousijn, Wallis, Harrison, & Nobre, 2014). Berger discovered alpha oscillation in 1929, and it has been a research topic in neuroscience ever since (Berger, 1929; Mierau, Klimesch, & Lefebvre, 2017). Previous studies have shown that alpha rhythms are mainly distributed in the parietal and occipital regions and are considered to be typical EEG waveforms of basal and resting EEG activity. Some research showed that source of alpha activity isolated in the fifth layer of the occipital cortex (Lopes Da Silva & Storm Van Leeuwen, 1977; Silva, Amitai, & Connors, 1991) and two key cellular thalamic mechanisms come together to generate locally synchronized alpha activity (Hughes & Crunelli, 2005). EEG, magnetoencephalography, and positron emission tomography studies have shown that alpha rhythm is mainly produced in the posterior brain region (Compston, 2010; Hari, Salmelin, Makela, Salenius, & Helle, 1997; Sadato et al., 1998). Alpha oscillations are generally present during relaxed and awake brains and are related to times of sensory and cognitive inhibition (Klimesch et al., 2007). They play an important and positive role in cognitive processing (Haegens et al., 2014) and display well‐defined developmental characteristics (Ingber & Nunez, 2011).

Some studies have reported significant reduction in alpha power in children with ASD compared to TD children (Shephard et al., 2018; Wang et al., 2013). Recent studies have found that the alpha peak frequency (APF) was very sensitive to the developmental changes of neural networks (Mierau et al., 2017), and APF increases with cognitive need and task participation. Significant individual differences were found in alpha frequency, and thus, the individual alpha peak frequency (iAPF) should be used to estimate a particular person's alpha rhythm range, giving more accurate downstream analysis results (Haegens et al., 2014). The cognitive ability of ASD children and TD children differ, suggesting that iAPF may be used as a biomarker. Studies have shown that the value of APF in ASD children was lower than TD children (Dickinson, DiStefano, Senturk, & Jeste, 2018). Therefore, how to accurately extract and analyze the alpha band is an important topic in the development of EEG biomarkers for ASD children.

There are many methods to extract rhythms from noisy signals. The Fourier transform is the most commonly used method to obtain required frequency components from signals of interest. However, it only applies to stationary signals and cannot reflect changes in the instantaneous frequency of the signal over time (Güven & Batbat, 2018). Empirical mode decomposition is also a widely used signal processing method that allows the separation of oscillation components contained in the signal using the inherent mode function (Liang et al., 2015). However, the extracted modes may lack clear frequency differences using intrinsic mode function, which might cause periodic signal distortion (Güven & Batbat, 2018). Wavelet transform can also be used to remove artifacts and decompose frequency components, but has relatively higher computational burden, and trouble separating overlapping components in time‐frequency space (Azami & Sanei, 2012). The singular spectrum analysis (SSA) method used in this study is an extensive method for nonlinear time series analysis. SSA can remove noise components from the EEG signal, as well as extract the desired rhythm (Mohammadi, Enshaeifar, Ghavami, & Sanei, 2015). It has been applied in other signal processing problems, such as noise detection in heart sounds (Sanei, Ghodsi, & Hassani, 2011), classification of open and closed eye states (Hu, Guo, Liu, & Wang, 2017), and classification of sleep and waking states (Mahvash Mohammadi, Kouchaki, Ghavami, & Sanei, 2016).

In this study, we applied SSA to the analysis of resting‐state EEG sequences recorded from children with ASD and TD children to eliminate noise and accurately to extract the alpha rhythm. We hypothesize that we may find biomarkers that could accurately distinguish between ASD and TD children through analysis and comparison of the alpha rhythm of ASD and TD children.

## 2 METHODS

### 2.1 Subjects

In this study, Gpower software was used for prior analysis according to alpha level. Statistical efficacy (1–*β*) and effect size were used to calculate sample size. The result showed that 27 subjects were needed in both two groups. We enrolled 46 children with ASD (23 males and 23 females; mean ± *SD* age: 4.1 ± 0.713 years) and 63 TD children (33 males and 30 females; mean ± *SD* age: 4.2 ± 0.453 years) in this experiment. There was no significant difference in age and gender between two groups. All children with ASD were diagnosed by professional psychiatrists according to Diagnostic and Statistical Manual of Psychiatry‐V criteria and the Children's Autism Rating Scale. Study inclusion criteria include participants with ASD and aged between 3 and 5 years. Exclusion criteria included a pacemaker or other metal device in the body, skull defects, and diagnosis of other mental disorders. All the TD children were recruited from a local kindergarten. Before the experiment, we described the entire procedure to children's parents and obtained their written informed consent. The experiments were approved by the Ethics Committee of Beijing Normal University.

### 2.2 EEG data collection

An eight‐channel EEG acquisition system was used to collect EEG data. The channels were placed at 10/20 locations F3, F4, Tp7, Tp8, P3, P4, O1, and O2. Cz acted as the reference electrode, and the impedances of electrodes were controlled below 50kΩ during data collection. The sampling frequency was 1,000 Hz. The participants were required to sit in a comfortable chair in a quiet environment with eyes open for about 5 min. In this study, two channels (O1, O2) in the occipital area were selected for analysis based on the work of Bazanova (Bazanova, 2011), which indicated that the optimal location for determining the iAPF range was located in the posterior region of the brain. The signals from these two channels were closely related to the alpha rhythm.

### 2.3 Data preprocessing

We used EEGLAB toolbox for noise removal. In this study, adaptive artifact detection was used to detect artifacts. This method segments the original time series. If there is an artifact component that exceeds a set threshold within a certain period of time, then this period of time is discarded. In this study, we divide the acquired EEG data into nonoverlapping time series of 4 s (Durka, Klekowicz, Blinowska, Szelenberger, & Niemcewicz, 2003). EMG, breathing, and other interfering signals were removed, and data were sampled to 200 Hz.

### 2.4 Adaptive SSA

Singular spectrum analysis can extract desired oscillations and eliminate noise components from time series data. We used SSA to extract the alpha rhythm from the noisy EEG time series data. The SSA method consists of two parts: decomposition and reconstruction.

The decomposition phase is divided into two parts: time‐delay embedding and singular value decomposition (SVD).

#### 2.4.1 Time delay embedding

In this step, the one‐dimensional EEG time series S=(S1,S2,…,SN) of length N is mapped onto a multi‐dimensional trajectory matrix *X*:

(1) $$X=\left(\begin{array}{cccc} S1 & S2 & \cdots & \mathrm{SK}\\ S2 & S3 & \ldots & SK+1\\ \vdots & \vdots & \ddots & \vdots \\ \mathrm{SL} & SL+1 & \cdots & \mathrm{SN} \end{array}\right),$$

where *L* is the length of the window. *K* = *N*–*L* + 1. *X* is a Hankel matrix, which repeats along the antidiagonal elements (Enshaeifar, Kouchaki, Took, & Sanei, 2016; Hu et al., 2017; Mees, Rapp, & Jennings, 1987).

#### 2.4.2 Singular value decomposition

The SVD is used to decompose the trajectory matrix into its feature subspace, which can be rewritten as:

(2) X=X1+X2+…+Xr.

We first calculate the covariance matrix *X* ^T^ *X* and obtain its *L* eigenvalues, placed in decreasing order (λ1≥λ2≥⋯≥λL≥0).$Xi=\sqrt{\lambda i}uiv_{i}^{T}$ represents the ith reconstruction component (RC), $u_{i}$(left singular vector) is the eigenvector of the covariance matrix *XX* ^T^, and $v_{i}$ (right singular vector) is the eigenvector of the covariance matrix *XX* ^T^. Each $uiv_{i}^{T}$ represents a feature of the EEG signal, and each eigenvalue represents the contribution of the corresponding feature to the original signal (Hu et al., 2017; Mahvash Mohammadi et al., 2016; Xu, Hu, Ji, & Wang, 2018).

The reconstruction phase consists of two parts, grouping and reconstruction (diagonal averaging). In the grouping step, the *X_i_* matrices are grouped into several groups, corresponding to different EEG rhythms and noise. The grouping rules depend on the requirements of the problem, characteristics of the signal, and characteristics of the noise (Hu et al., 2017). The unwanted groups, such as those containing noise, are discarded. The time series is then reconstructed from the remaining *X_i_* matrices in the diagonal averaging step. The reconstructed signal is used for subsequent analysis.

In the decomposition phase, RCs are obtained with corresponding eigenvalues. The sorted eigenvalues decay rapidly, that is, the RCs corresponding to the largest few eigenvalues dominate the EEG time series. Signal‐related eigenvalues with high energy are located in the lower subspace, while weaker signals appear in the higher subspace. In our EEG analysis, electrooculograph (EOG) signals, caused by eye blinks and ocular movements, were the main artifact. They are large amplitude, low frequency potential shifts. In this study, when the amplitude of the EEG signal was high, the components corresponding to the largest two eigenvalues were removed. Otherwise, the component corresponding to the largest eigenvalue was removed (Teixeira, Tome, Lang, Gruber, & Martins da Silva, 2006).

After the artifact removal procedure above, the alpha rhythm was extracted from the EEG time series. The rhythm is the oscillation component of the EEG time series, including but not limited to the periodic component. The RCs are divided into periodic and aperiodic RCs. RCs with similar eigenvalues belong to the same periodic component (PC) (Vautard, Yiou, & Ghil, 1992), where the periodic component eigenvalue similarity criteria is:

(3) $$\left|1‐\frac{\lambda j}{\lambda i}\right|<K$$

The value of K is chosen according to the amplitude of the waveform; because alpha rhythm contains high amplitude, *K* = 0.05 was chosen (Mahvash Mohammadi et al., 2016).

Using the Fourier transform, the peak frequency *f*_max_ of an RC (or PC) is found by:

(4) $$f_{\max}=\underset{f}{\arg\max}\left\{abs[FFT(RC)]\right\},$$

where FFT(RC) is the fast Fourier transform of RC. RCs and PCs with the same peak frequencies are grouped, together corresponding to a brain rhythm (Hu et al., 2017).

### 2.5 Rhythmic absolute energy

#### 2.5.1 Frequency domain method

The power spectrum *P*(*f*) is calculated from the EEG. If the boundaries of the alpha rhythm band are *a* to *b*, the alpha rhythm frequency domain absolute energy is:

(5) $${\text{ab}}_{\alpha }1=10\times \sum_{i=a}^{b}p_{i}^{\alpha }$$

#### 2.5.2 Time‐domain method

Given a single‐channel EEG signal S=(S1,S2,…,SN), using SSA to extract the alpha rhythm time domain signal x=(x1,x2,x3…xn), the alpha rhythm time domain relative energy is:

(6) $${\text{ab}}_{\alpha }2=10\times \sum_{i=1}^{n}x_{i}^{2}$$

Alpha peak frequency.

The APF was found using the center of gravity (CoG) frequency, that is, “weighted sum of spectral estimates divided by frequency band power” (Christie, di Fronso, Bertollo, & Werthner, 2017; Klimesch, 1999):

(7) $$\text{COG}=\frac{\sum_{f=n}^{m}P(f)\times f}{\sum_{f=n}^{m}P(f)}$$

Here, *n* is the low frequency threshold, *m* is the high frequency threshold, and *P*(*f*) is the energy at frequency *f*. We determined the alpha rhythm range to be 0.8* × CoG* ~1.2 × *CoG*. This method is more sensitive to the entire shape of the alpha peak than visual inspection for the highest local peak in the spectrum (Klimesch et al.,). In this study, *n* = 7 Hz, *m *= 14 Hz. We use this method since the frequency of the alpha rhythm has been shown to be wider than the conventional narrowband fixed bandwidth (8–12 Hz). This method can provide a more accurate peak estimate, especially if multiple peaks are identifiable (Christie et al., 2017; Haegens et al., 2014).

### 2.6 Classification

We use support vector machines (SVM) and area under the receiver operating characteristic (ROC) curve (AUC) to perform classification and classifier evaluation, respectively. SVM has been widely used for its excellent classification performance (Luo, Liu, Yap, Liedberg, & Ser, 2018), which can find a class‐separating hyperplane in feature space that maximizes the margin between data points and the hyperplane (Cover, 1965). The determination of the classification hyperplane depends on the closest data points, which are called support vector (Mueller, Candrian, Kropotov, Ponomarev, & Baschera, 2010). It contains a variety of SVM classifiers. In this study, a linear SVM classifier was selected (Basu & Xavier Savarimuthu, 2002). To evaluate classification models, cross‐validation of the data is required. K‐fold cross‐validation is used to randomly divide sample into K subsamples of similar size, one of which is used as a test set, and remaining K‐1 subsamples are used for training. K subsamples are used as test sets in turn, and we get k classification accuracy. Then, average them and get a classification accuracy. Common 10‐fold cross‐validation was used in this study.

Receiver operating characteristic curve (ROC) refers to the curve drawn by different results obtained by different judgment standards under stimulus conditions and has been widely used in the accuracy of diagnostic tests. The ordinate of the ROC curve is the true positive rate (TPR), which refers to the ratio that is correctly judged to be classified as positive in the samples that are actually positive; the abscissa is the negative positive rate (FPR), which refers to the ratio of false‐positive classifications in samples that are actually negative. Because the horizontal and vertical coordinates of ROC curve have no correlation, we can only consider ROC curve as infinite number of points, and not as a function curve. When evaluating the model, the index used is the area under the curve (Hanley & Mcneil, 1982). Area under the curve (AUC) is the area under the ROC curve, which means that if a positive and a negative sample are randomly selected, the probability that the classifier correctly judges the positive sample is higher than selecting negative sample. Subjectivity can be avoided during threshold selection (Fawcett, 2006). The value of AUC is between 0 and 1, and the higher the accuracy of the classifier. In this study, AUC value is used as the index of the model.

## 3 RESULTS

**Figure 1:**
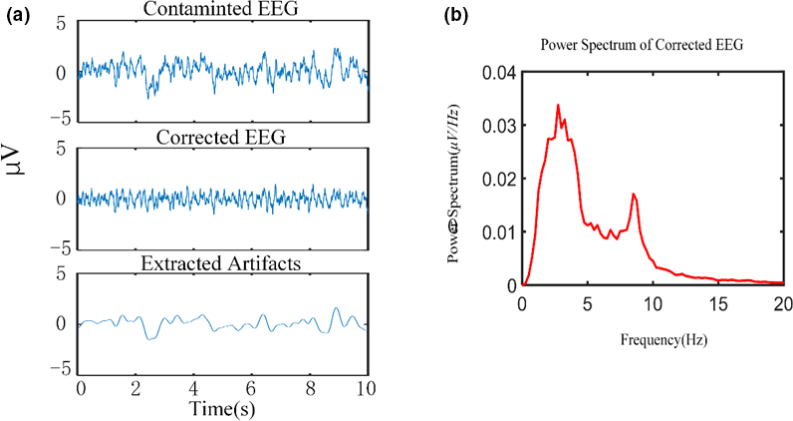
a) Contaminated EEG, corrected EEG, and extracted artifacts; b) Spectrum of EEG processed by SSA, removing low frequency EOG signals, showing the desired alpha rhythm

**Figure 2:**
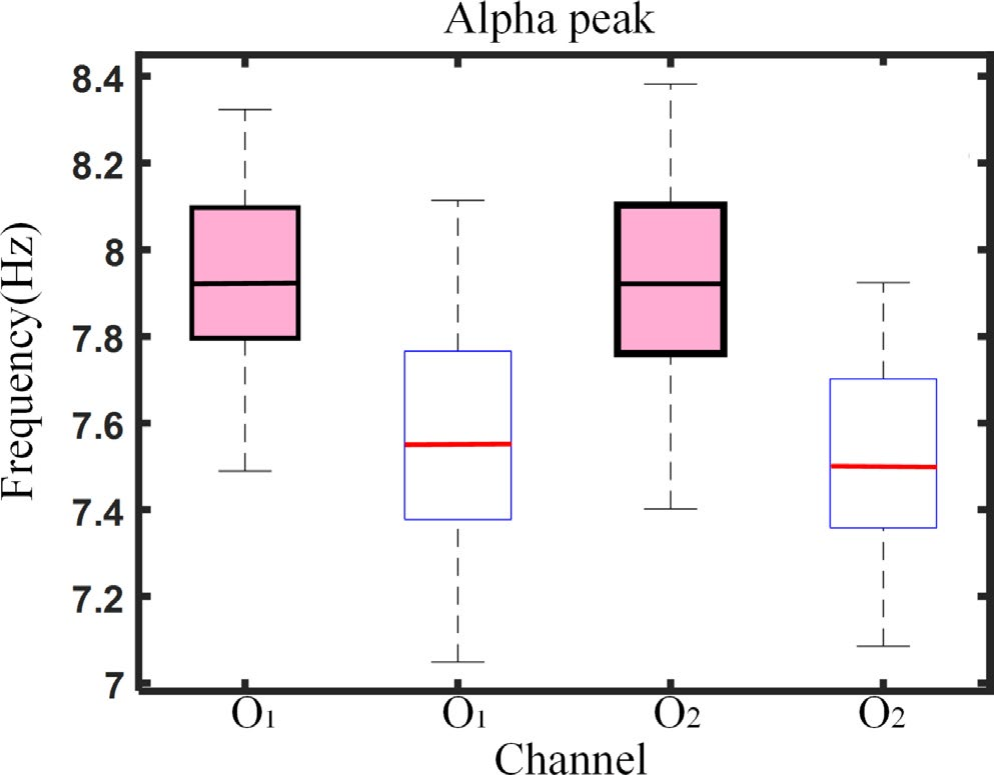
Alpha peak frequency at electrodes O1, O2 in TD and ASD children. Alpha peak frequency of TD children was significantly higher than that of children with ASD. 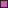 TD; 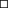 ASD

**Figure 3:**
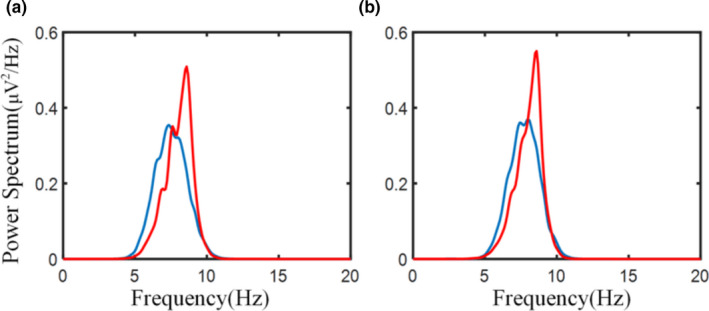
Alpha rhythm in children with ASD and TD children: power spectra of the extracted alpha rhythm from the electrode at a) O1 and b) O2. 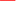 TD; 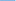 ASD

1. Adaptive SSA can effectively remove the influence of artifacts from the signal and extract the desired alpha rhythm. The cleaned EEG signal is shown in Figure 1a, and its spectrum is shown in Figure 1b. Using the time‐domain method to calculate the absolute energy of the alpha rhythm can improve classification accuracy. The alpha absolute energy calculated by the time‐domain method of Equation 6 (named ABP#2) led to a classification accuracy of 87.16% and an AUC value of 0.9037. The alpha absolute energy calculated by the frequency domain method of Equation 5 (ABP#1) led to a classification accuracy of 83.49% and an AUC value of 0.8903 (Table 1).
2. The peak frequency of the alpha rhythm in children with ASD is significantly lower than that of TD children. Previous work found that alpha peak frequency of TD children gradually increased with age, and the alpha peak frequency of ASD children was lower than that of TD children (Dickinson et al., 2018). To explore the iAPF of children with ASD in our young sample, Equation 7 was used separately for data from the O1 and O2 electrodes. The results are shown in Figure 2. The mean (M) and standard deviation (*SD*) of iAPF values for TD children were *M_O1_* = 7.9223, *SD_O1_* = 0.21; *M_O2_* = 7.9059, *SD_O1_* = 0.23. For children with ASD, iAPF values were *M_O1_* = 7.5723, *SD_O1_* = 0.24; *M_O2_* = 7.5120, *SD_O1_* = 0.23. The iAPF values at O1 and O2 in the ASD group were significantly lower than that of the TD group (*t* tests, O1: *t* = −8.138, *p *< .0001; O2: *t *= −8.812, *p *< .0001).
3. The absolute energy and peak frequency of the alpha rhythm can be used as features to distinguish between children with ASD and TD children. Alpha peak frequency differences lead to differences in the calculated alpha band range. According to the iAPF, children's alpha band range was less than 8–12 Hz and the frequency ranges of TD children and children with ASD were different. Thus, an individualized alpha band range was used for spectral estimation. SSA was used to extract the individualized alpha rhythm according to the iAPF. Based on a previous study by Xu and colleagues (Xu et al., 2018), the SSA parameters *L *= 60, *C *= 12 were used in this study, and C represents the number of selected RC. The desired alpha rhythm could be effectively extracted from the reconstructed components, as shown in Figure 3. The absolute energy of a channel's individualized alpha rhythm (iABP) was calculated by the time‐domain method (Equation 6) and used as a feature for the classifier. The classification accuracy was 81.65%, and the AUC value was 0.9023 (Table 1). The classification performance was lower compared to ABP#2, indicating that the range of alpha band selected according to traditional alpha band concept was not suitable for extracting alpha rhythm of young children with developmental disabilities.

**Table 1:** SVM classification results for different features

	Accuracy	AUC
ABP#1	83.49%	0.8903
ABP#2	87.16%	0.9037
iABP	81.65%	0.9023
iAPF	75.23%	0.8571
iABP + iAPF	92.66%	0.9752

Subsequently, we attempted to classify using the iAPF. The classification accuracy using iAPF calculated via *CoG* was 75.23% (Table 1). Although the iAPF of children with ASD and TD children was significantly different, it was not enough to use iAPF alone to distinguish two groups with high accuracy.

The absolute energy and alpha peak frequency of the individualized alpha rhythm in children with ASD were lower than TD children, but when these features were used for classification separately, the classification accuracy was not satisfactory. Therefore, we combined the individualized alpha peak frequency with the individualized alpha absolute power (iABP + iAPF) as features for the SVM classifier. The resulting classification accuracy was 92.66%, and the AUC value was 0.9752.

## 4 DISCUSSION

Previous studies have found that SSA can extract and eliminate noise components from signals, extract periodic signals in EEG time series, and extract desired brain rhythms (Hu et al., 2017; Mahvash Mohammadi et al., 2016; Mohammadi et al., 2015; Sanei et al., 2011). Researchers have used SSA to detect murmurs from monophonic heart sound signals for accurate separation (Sanei et al., 2011). Hu et al. proposed an adaptive SSA method to extract the alpha rhythm and distinguish between EEG data from open‐ and closed‐eyes states and verified the effectiveness of the method (Hu et al., 2017). In this study, we applied SSA to analyze EEG signal recorded from children with ASD and TD children to eliminate noise and accurately extract the alpha rhythm and also obtained satisfied results.

Some studies have found decreases in alpha rhythm power spectrum in children with ASD compared with TD children (Han et al., 2018; Shephard et al., 2018; Wang et al., 2013). We used SSA for alpha rhythm extraction from EEG recorded from children with ASD. Since the adaptive SSA method removed low frequency noise such as EOG, according to magnitude of the signal amplitude, we could also use absolute power as an index to reduce individual differences in the power spectrum analysis process. Our results showed that the adaptive SSA method could effectively extract the alpha rhythm. Consistent with previous studies, we found that the absolute energy of the alpha rhythm in children with ASD was lower than that of TD children. Decreased alpha energy means a lack of alpha inhibition and inefficient neurological function, which may be due to cognitive impairments in most ASD children. Thus, alpha rhythm absolute energy might be used as a biomarker to assist in the diagnosis of ASD.

We found that the APF of children with ASD was significantly lower than that of TD children, which was consistent with previous findings (Dickinson et al., 2018; Haegens et al., 2014; Mierau et al., 2017). Our findings indicated the significant difference of APF between ASD and TD children, especially in occipital region, suggesting spatial specificity of APF is important for discriminating between groups. APF has been shown to vary with personal cognitive involvement in task performance, and good performance was associated with increased APF, but a drop in performance are related to decrease in APF (Ng & Raveendran, 2007). Therefore, characteristic and development of brain can be indicated by APF, which can also be used to distinguish the differences between ASD and TD children. These converging lines of evidence support the hypothesis that children with ASD are different from TD children in brain development and behavioral performance. The evidences also support the hypothesis that children with ASD differ from TD children in brain development and behavior. These findings can also help us understand ASD and the indicators could be used to assist in the diagnosis of ASD.

Although previous studies have reported abnormalities in the absolute energy and APF in children with ASD (Dickinson, DiStefano, Senturk, & Jeste, 2018; Edgar et al., 2019), no studies have combined iABP and iAPF. Our results showed when combined absolute energy and APF at occipital region, we can obtain better classification accuracy, which may assist in the diagnosis of ASD. In our study, the frequency range of alpha rhythm was determined by its peak frequency. Regarding the use of fixed alpha band versus individualized alpha band, we believe that when individualized differences are taken into account, better classification results can be obtained. ASD is a very complex disorder; therefore, combination of multiple features may lead to a more accurate result. Furthermore, individualized alpha band is more suitable for children with ASD because their brain development process is different from TD children.

There are some limitations in our study. The sample size was small, so we could not quantify the method used in this study was sensitive with increase of age or not. We plan to enroll more children to verify the effectiveness. Because ASD children cannot cooperate well, the number of EEG channels is little, and more channels can be tried later, making the results more accurate.

## 5 CONCLUSION

Our study found that individualized relative alpha energy metabolism in the occipital lobe region and the individualized alpha frequency of children with ASD were significantly lower than TD children, indicating that the relative energy of alpha rhythms and the lower alpha frequency of children were clinically significant. The decline in cognitive function is consistent, reflecting that the brain development of children with ASD may be slow. Combining iABP and iAPF here gives a classification accuracy of 92.66%, so it can provide a scientific and objective basis for the auxiliary diagnosis of children with ASD.

## CONFLICT OF INTEREST

All authors declared that they have no conflicts of interest to this work.

## AUTHOR CONTRIBUTIONS

Xiaoli Li contributed to the conception of the study; Jiannan Kang contributed significantly to analysis and manuscript preparation; Jiajia Song performed the data analyses; and Jie Zhao performed the analysis with constructive discussions. All authors have read and approved the content of the manuscript.

## Data Availability

The data that support the findings of this study are available from the corresponding author upon reasonable request.
